# Synthesis and Biological Evaluation of Unsymmetrical Curcumin Analogues as Tyrosinase Inhibitors

**DOI:** 10.3390/molecules18043948

**Published:** 2013-04-03

**Authors:** Yongfu Jiang, Zhiyun Du, Guihua Xue, Qian Chen, Yujing Lu, Xi Zheng, Allan H. Conney, Kun Zhang

**Affiliations:** 1 Institute of Natural Medicine & Green Chemistry, School of Chemical Engineering and Light Industry, Guangdong University of Technology, Guangzhou 510006, China; 2 Department of Chemistry, Zhejiang Normal University, Jinhua 321004, China; 3 State Key Laboratory of Natural and Biomimetic Drugs, Peking University, Beijing 100191, China

**Keywords:** unsymmetrical curcumin analogues, synthesis, tyrosinase inhibitors, biological evaluation, inhibition kinetics

## Abstract

Synthesis and biological evaluation of unsymmetrical curcumin analogues (UCAs) have been achieved. Tyrosinase inhibitory activities were found for most of the prepared synthetic UCAs. Among them, compounds containing 4-hydroxyl-substituted phenolic rings with *C*-2/*C*-4- or *C*-3/*C*-4-dihydroxyl-substituted diphenolic rings were more active (IC_50_ = 1.74~16.74 μM) than 4-butylresorcinol and kojic acid, which suggested that the 4-hydroxyl groups in UCAs play a crucial role in tyrosinase inhibitory activities. The inhibition kinetics analyzed by Lineweaver-Burk plots revealed compounds **3c** and **3i** containing catecholic rings were mixed-competitive inhibitors, whereas compounds **3d** and **3j** containing resorcinolic rings were competitive inhibitors. The preliminary evaluation results of acute toxicity showed the representative **3d** and **3j** were non-toxic in mice dosed at 1,200 mg/kg. This research suggests that, with the advantage of being readily prepared small molecules, polyphenolic UCAs have the potential to develop into pharmacological inhibitors of tyrosinase.

## 1. Introduction

Generally, human skin color is determined by the type and the amount of melanin, a natural pigment produced by melanocytes, which plays a crucial role against skin photocarcinogenesis. However, abnormal melanin pigmentation can cause dermatological disorders such as lentigo, age spots, melasma, ephelide and senile lentigines [1,2]. Tyrosinase [EC1.14.18.1] is a key enzyme of the rate-limiting step for the biosynthesis pathway of melanin pigment. Tue to their potent activities tyrosinase inhibitors have been of particular interest both skin medications [3,4,5] and cosmetics [6,7] to prevent hyperpigmentation. Recently, various naturally occurring or synthetically produced tyrosinase inhibitors have been reported [2,8,9,10,11,12]. However, most of them suffered from limitations such as low activity, high toxicity and insufficient penetrative ability [13,14].

Curcumin, an antioxidant polyphenol from the rhizome of *Curcuma longa* Linn, is a major ingredient of turmeric, and it has been used for the therapy of inflammatory and infectious diseases in ayurvedic medicine [15]. Many studies showed that curcumin had cancer preventive [16], anti-inflammatory [17], antioxidative [18] and antiviral activities [19]. In addition, the safety of curcumin is evident by its consumption for centuries at levels up to 100 mg/day by people in some countries [20]. These beneficial properties have attracted numerous efforts for the development of curcumin as a safe therapeutic agent. Curcumin has been approved as a natural yellow color additive and antioxidant in cosmetics for many years [21]. Recently, curcumin was demonstrated to be beneficial to treat some skin diseases [22,23], and one of its derivatives, tetrahydrocurcumin, was recommended to be used in cosmetics as a lighting agent [24]. Furthermore, Lee *et al* recently reported that some curcumin analogues exhibited inhibitory activity against tyrosinase [25]. These reports attracted our interest to further study the inhibitory effect of curcumin analogues on tyrosinase. 

**Scheme 1:**
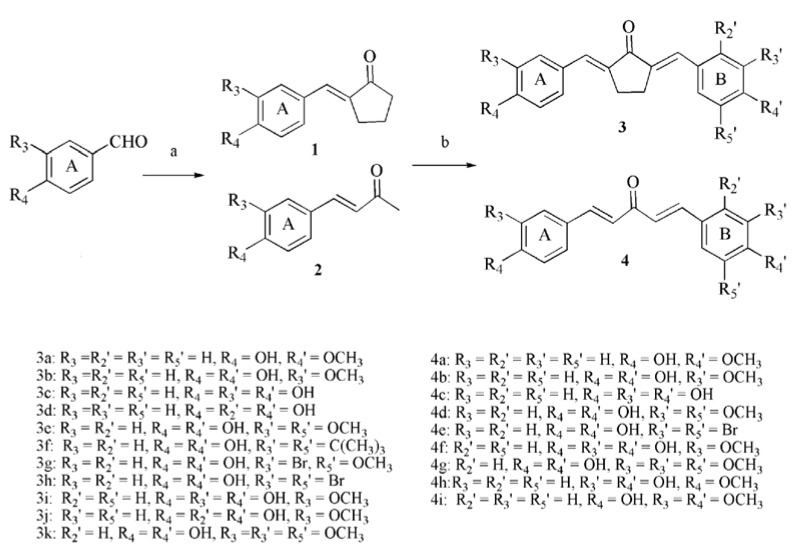
Synthesis route of polyphenolic unsymmetrical curcumin analogues.

In this study, considering polyphenolic compounds exhibited potent inhibitory activity against tyrosinase, and their 4-hydroxyl groups played a crucial role in some tyrosinase inhibitors [26,27,28], a series of unsymmetrical curcumin analogues (UCAs) bearing 4-hydroxyl groups were synthesized (Scheme 1) and their inhibitory activities against tyrosinase were evaluated. In addition, the inhibition mechanism and acute toxicity of several potent UCAs were also investigated in order to achieve our aims of developing novel tyrosinase inhibitors with potent activities and lower side effects.

## 2. Results and Discussion

### 2.1. Chemistry

The syntheses of polyphenolic UCAs (compounds **3a**–**k** and **4a**–**i**, Scheme 1) were easily completed by a facile two-step sequence without the need for hydroxyl group protection. This began with an aldol condensation of an aromatic aldehyde (4-hydroxybenzaldehyde or 4-hydroxy-3-methoxy-benzaldehyde) with excess acetone or cyclopentanone under basic conditions (aq. NaOH) to afford a conjugated enone **1** or **2**. Finally, one more aldol condensation of a different aromatic aldehyde (using various substituted benzaldehyde derivatives) with the corresponding intermediate **1** or **2** under acidic conditions (catalytic amount of conc. HCl) gave the desired UCAs **3** or **4** in 50~75% yield over two steps. Thus, twenty polyphenolic UCAs containing 4-hydroxyl groups on ring A were prepared. Among them, hydroxyl groups are only present at the *C*-4 position on ring A, while there are *C*-4 hydroxyl groups for monophenols and *C*-2/*C*-4 or *C*-3/*C*-4 dihydroxyl groups for diphenols (catecholic or resorcinolic rings) on ring B.

### 2.2. Inhibitory Effects on the Diphenolase Activity of Mushroom Tyrosinase

**Figure 1:**
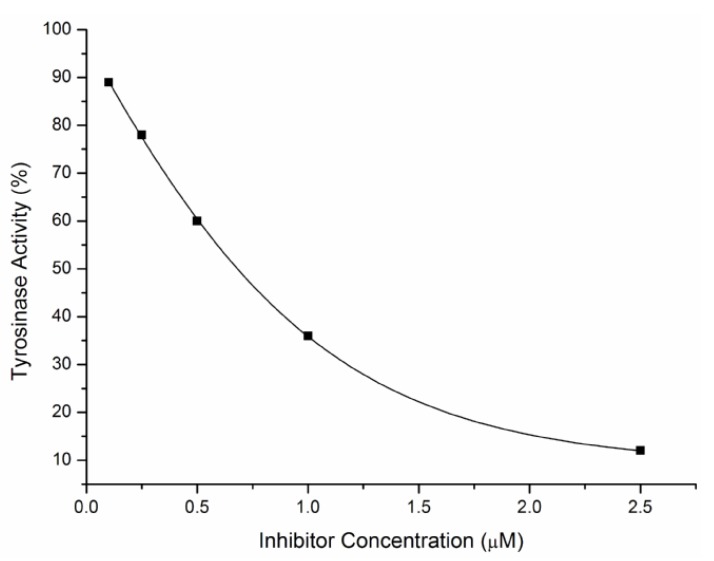
Effect of compound **3d** on the diphenolase activity against mushroom tyrosinase for the catalysis of l-DOPA at 25 °C.

UCAs **3a**–**k**, **4a**–**i** were subjected to tyrosinase inhibitory assays by measuring the oxidation of l-DOPA according to the literature protocol [26]. For example, the effect of inhibitor **3d** on the diphenolase activity against mushroom tyrosinase was outlined in Figure 1. It was illustrated that the course of enzyme activity inhibition by the inhibitor was concentration-dependent. With the increase of the concentration of the inhibitor, the activity of the remaining enzyme decreased rapidly. Enzyme inhibition data were expressed as IC_50_ and values are summarized in Table 1. 4-Butylresorcinol and kojic acid were used as the positive controls. As shown in Table 1, most of synthetic polyphenolic UCAs (compounds **3b**–**e**, **3i**, **3j**, **4b**–**d**, **4f** and **4h**) displayed <100 μM inhibitory activities against mushroom tyrosinase. To our delight, UCAs **3c**, **3d**, **3i**, **3j**, **4c** and **4f** containing dihydroxyl groups on ring B exhibited significant inhibitory activities (1.74~16.74 μM). It is noteworthy that **3d** is the strongest inhibitor, with an IC_50_ value of only 1.74 μM, which was about 16-fold and 6-fold lower than that of kojic acid (28.59 μM) and 4-butylresorcinol (11.27 μM), respectively.

We then turned to our analysis of the inhibitory Structure Activity Relationship (SAR). To evaluate the influence of the hydroxyl positions of UCAs, without change of ring B containing dihydroxyl groups, when the 4-hydroxyl group on ring A of compound **4c** (IC_50_ = 4.64 μM) was replaced by a methoxyl group, the activity of the corresponding compound **4h** (IC_50_ = 86.92 μM) decreased significantly (>16-fold difference). This result suggested the hydroxyl groups at the 4-position on ring A of UCAs played a key role in the tyrosinase inhibitory activity. As mentioned above, compounds containing catecholic or resorcinolic ring displayed higher inhibitory activities, while compounds containing resorcinolic rings (**3d** and **3j**) were more active than compounds containing catecholic rings (e.g., **3c**, **3i** and **4f**). Other substituents on ring A or B also influenced the inhibitory activities of UCAs. For instance, when the hydroxyl group at *C*-3 on ring B of **3c** (IC_50_ = 6.78 μM) was switched to a methoxyl group, the activity of the corresponding **3b** (IC_50_ = 56.64 μM) decreased (>8-fold difference). When a bromine substituent was introduced into ring B, none of the bromides (**3h**, **3g** and **4e**) showed activities (>170 μM). The bulky *tert*-butyl substituent on ring B also decreased the activity (**3f**, IC_50_ = 168.36 μM). Interestingly, introduction of an additional methoxyl group into ring B of **3b** led to a much more active compound **3e** (IC_50_ = 9.66 μM).

**Table 1:** Inhibitory effects of UCAs against mushroom tyrosinase.

Compound	IC_50_ (μM)	Compound	IC_50_ (μM)
**3a**	189.42	**4a**	182.86
**3b**	56.64	**4b**	46.24
**3c**	7.78	**4c**	4.64
**3d**	1.74	**4d**	7.20
**3e**	9.66	**4e**	>200
**3f**	168.36	**4f**	16.34
**3g**	173.48	**4g**	>200
**3h**	>200	**4h**	86.92
**3i**	16.74	**4i**	>200
**3j**	2.78	4-Butylresorcinol	11.27
**3k**	>200	Kojic acid	28.59

### 2.3. Kinetic Analysis of Selected Compounds on Mushroom Tyrosinase

**Figure 2:**
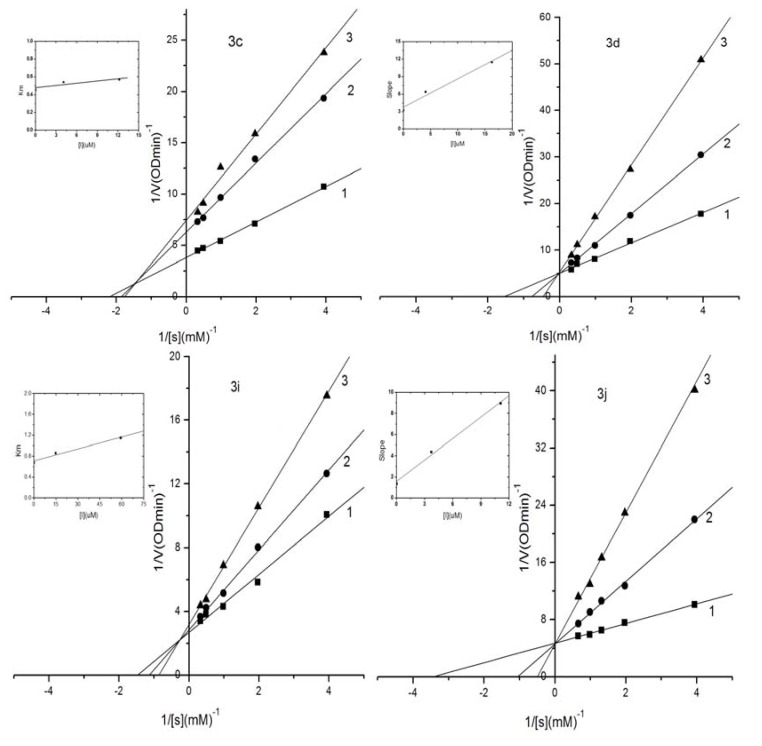
Lineweaver–Burk plots for inhibition of compounds **3c**, **3d**, **3i** and **3j** against mushroom tyrosinase for the catalysis of l-DOPA. Concentrations of **3c**, **3d**, **3i** and **3j** for curves 1–3 were 0.0 μM, 4.06 μM, 12.18 μM; 0.0 μM, 4.06 μM, 16.23 μM; 0.0 μM, 14.8 μM, 59.2 μM; 0.0 μM, 3.70 μM, 11.09 μM, respectively.

The inhibitory mechanism of selected compounds **3c**, **3d**, **3i** and **3j** against mushroom tyrosinase during the oxidation of l-DOPA was determined by the same methods. Double-reciprocal plots of the inhibition kinetics of selected compounds against tyrosinase are shown in Figure 2. Compounds **3c** and **3i** containing *o*-dihydroxyl groups on ring B were mixed-competitive inhibitors, as illustrated in Figure 2 where increasing the concentration of **3c** or **3i** resulted in straight lines with different slopes and intercepts, while the lines were intersected in the second quadrant. However, **3d** and **3j** containing *m*-dihydroxyl groups on ring B were competitive inhibitors because the intersection of those straight lines was on the Y-axis.

### 2.4. Evaluation of Acute Toxicity in Mice

**3d** and **3j** were used as the representative compounds to evaluate the toxicity in mice. The experimental results demonstrated that daily dose of 1,200 mg/kg in mice led to no mortality for each mouse, and the observation of characteristics of mice in the control and treated groups, such as food intake, water drinking, body weight, general appearance, skin and fur, eyes and nose, respiration, urine, feces, locomotor, were not abnormal (Table 2). The autopsy results of all mice at the end of the experimental period (14 days) also revealed that no apparent changes were detected in any mice organs. These results indicated that **3d** and **3j** had no acute toxicity in mice at a dose of 1,200 mg/kg.

**Table 2:** Observation of acute toxicity in mice for compound **3d**.

Item	Control		Treated by 3d		Treated by 3j	
	Male	Female	Male	Female	Male	Female
Food Intake (g/day)	5.1 ± 0.3	3.9 ± 0.4	5.1 ± 0.5	4.0 ± 0.4	5.2 ± 0.4	3.9 ± 0.5
Drinking Water (mL/day)	5.5 ± 0.7	4.2 ± 0.4	5.6 ± 0.6	4.2 ± 0.5	5.4 ± 0.5	4.1 ± 0.4
Body Weight (g)	31.3 ± 2.5	27.1 ± 2.3	30.9 ± 2.6	27.3 ± 2.9	30.1 ± 2.8	26.9 ± 2.1
General Appearance	√	√	√	√	√	√
Skin and Fur	√	√	√	√	√	√
Eyes, Nose	√	√	√	√	√	√
Respiration	√	√	√	√	√	√
Urine	∆	∆	∆	∆	∆	∆
Feces	∆	∆	∆	∆	∆	∆
Locomotor	√	√	√	√	√	√

Note: √ stands for Normal, and ∆ stands for No Discoloration. CD-1 mice (9 males and 9 females; 7~8 weeks old) were divided into 6 equal groups (controls: male, female, treated by 3d: male and female, treated by 3j: male and female). Mice had free access to distilled water and commercial standard diet. Food Intake and Drinking Water were measured daily and averaged statistically, and Body Weight was measured in the last day. General Appearance, Skin, Fur, Eyes, Nose, Respiration, Urine, Feces and Locomotor were observed daily.

### 2.5. Molecular Docking Study

Recently, the crystallographic structure of tyrosinase has been revealed. The three-dimensional structure of tyrosinase enables us to gain a better understanding of the tyrosinase inhibition mechanism. Considering compounds **3c**/**3i** and **3d**/**3j** exhibited different inhibition mechanisms, we selected the representative compounds **3c** and **3d** to study the interaction mode by docking.

**Figure 3:**
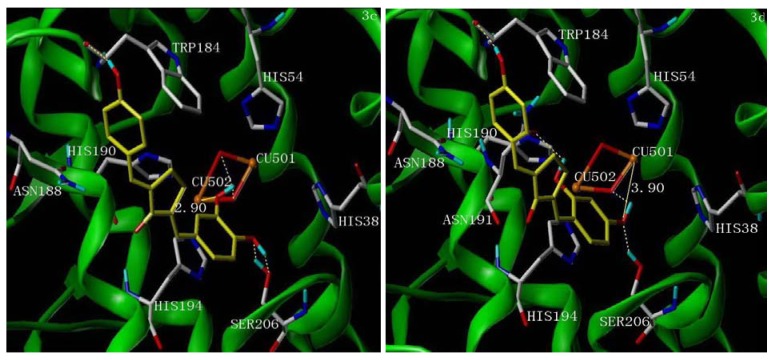
The proposed binding modes of **3c** (left picture) and **3d** (right picture) in the active site of tyrosinase (PDB access code 2ZWE). The inhibitor molecules are colored in yellow for carbon atoms. The dashed lines show hydrogen-bonding, the real lines show the distance of metal-coordination interactions. The docking models are generated using Surflex-Dock.

Although the structure of mushroom tyrosinase has not been determined, according to the reported crystallographic data, there is a high homology for the active center of most tyrosinases of different origin. In Figure 3, it was found that both **3c** and **3d** formed π-π stacks between ring B of the inhibitors and HIS194 of tyrosinase, and there were hydrogen bonds between the 4-position phenolic groups of **3c**/**3d** and the TRP184 residue of the active site of tyrosinase. This result further confirmed that hydroxyl groups in the 4-position of UCAs play a crucial role in the tyrosinase inhibitory activity.

The 4'-OH of **3c** formed two hydrogen bonds with SER206 and the 3'-OH of **3c** formed two hydrogen bonds with hydration water molecules of the binuclear copper ions of tyrosinase, while the distance between 3'-OH and copper ions CU502 was 2.90 Å (Figure 3, left). The 4'-OH of **3d** formed a hydrogen bond with SER206 and another hydrogen bond with one hydration water molecule of the binuclear copper ions, while the distance between the 4'-OH and copper ion CU501 was 3.90 Å (Figure 3, right), which is much weaker than that of **3c**. Furthermore, the 2'-OH of **3d** formed another hydrogen bond with ASN191.

## 3. Experimental

### 3.1. Reagents and General Procedures

Melting points were determined on a Yanagimoto micro-melting apparatus and were uncorrected. The ^1^H-NMR spectra (300 MHz) were measured on a Varian Gemini-2000 spectrometer using DMSO-*d*_6_as solvent and TMS as an internal standard. Chemical shifts were expressed in ppm units. Multiplicities were recorded as s (singlet), brs (broad singlet), d (doublet), t (triplet), q (quartet), m (multiplet). Mass spectra were obtained on a LC-MS-2010A spectrometer with ESI. Elemental analyses were performed on a Perkin Elmer 240C instrument. Thin-layer chromatography (TLC) was performed on Merck silica gel plates (DC-60 F254).

### 3.2. Synthesis

The appropriate substituted benzaldehyde (0.01 mol) was dissolved in a mixture of aqueous NaOH [8% (w/w), 16 mL] and ethanol (10 mL). The solution was then added dropwise to a stirred solution of acetone or cyclopentanone (0.03 mol) in aqueous NaOH [8% (w/w), 4 mL] at room temperature. The mixture was stirred at room temperature for 24 h. The precipitation was collected by filtration, and the solid was then dissolved in a mixture solution of water and ethanol. The solution was neutralized by 10% HCl in an ice-water bath to produce light yellow solid. The crude product was then filtered and recrystallized in ethanol to give the corresponding intermediate **1** or **2**.

The intermediate **1** or **2** (0.005 mol) and the appropriate substituted benzaldehyde (0.005 mol) were dissolved in a small amount of ethanol or THF solvent. Concentrated HCl (0.1 mL) was added and the mixture was stirred at room temperature for 0.5 h. The reactant was then placed in a sealed container for 2~3 days and tracked by TLC until the reaction completed. The precipitation was collected by filtration, washed by cool ethanol and distilled water and recrystallized in an appropriate solvent or purified using silica gel flash chromatography to give UCAs **3** or **4**.

*2-(4-Hydroxybenzylidene)-5-(4-methoxybenzylidene)cyclopentanone* (**3a**). Yield = 75%; m.p.: 241–243 °C; ^1^H-NMR δ = 10.02 (br, 1H), 7.63 (d, *J* = 8.7 Hz, 2H), 7.52 (d, *J* = 8.7 Hz, 2H), 7.34 (d, *J* = 6.6 Hz, 2H), 7.03 (d, *J* = 8.7 Hz, 2H), 6.86 (d, *J* = 8.7 Hz, 2H), 3.90 (s, 3H), 3.03 (s, 4H); ESI-MS: *m/z* = 304.9 (M^+^−H); Anal. Calc. for C_20_H_18_O_3_: C, 78.41; H, 5.92. Found: C, 78.36; H, 5.95. 

*2-(4-Hydroxy-3-methoxybenzylidene)-5-(4-hydroxybenzylidene)cyclopentanone* (**3b**). Yield = 72%; m.p.: 278–280 °C; ^1^H-NMR δ = 10.01 (brs, 1H), 9.62 (brs, 1H), 7.52 (d, *J* = 8.7 Hz, 2H), 7.33–7.32 (m, 2H), 7.22 (s, 1H), 7.16–7.13 (m, 1H), 6.86 (dd, *J* = 8.4, 2.7 Hz, 3H), 3.82 (s, 3H), 3.03 (s, 4H); ESI-MS: *m/z* = 320.8 (M^+^−H); Anal. Calc. for C_20_H_18_O_4_: C, 74.52; H, 5.63. Found: C, 74.49; H, 5.65.

*2-(3,4-Dihydroxybenzylidene)-5-(4-hydroxybenzylidene)cyclopentanone* (**3c**). Yield = 65%; m.p.: >300 °C; ^1^H-NMR δ = 10.00 (br, 1H), 9.53 (brs, 1H), 9.19 (brs, 1H), 7.51 (d, *J* = 8.7 Hz, 2H), 7.30 (s, 1H), 7.23 (s, 1H), 7.09 (d, *J* = 1.5 Hz, 1H), 7.00–6.97 (m, 1H), 6.87 (s, 1H), 6.84–6.80 (m, 2H), 3.00 (s, 4H); ESI-MS: *m/z* = 306.8 (M^+^−H); Anal. Calc. for C_19_H_16_O_4_: C, 74.01; H, 5.23. Found: C, 73.97; H, 5.24.

*2-(2,4-Dihydroxybenzylidene)-5-(4-hydroxybenzylidene)cyclopentanone* (**3d**). Yield = 68%; m.p.: 241–243 °C; ^1^H-NMR δ = 10.01 (brs, 1H), 9.97 (brs, 1H), 9.84 (brs, 1H), 7.73 (s, 1H), 7.50 (d, *J* = 8.7 Hz, 2H), 7.39 (d, *J* = 8.7 Hz, 1H), 7.28 (d, *J* = 2.1 Hz, 1H), 6.85 (d, *J* = 8.4 Hz, 2H), 6.42–6.30 (m, 2H), 3.00 (s, 4H); ESI-MS: *m/z* = 306.9 (M^+^−H); Anal. Calc. for C_19_H_16_O_4_: C, 74.01; H, 5.23. Found: C, 73.95; H, 5.25.

*2-(4-Hydroxy-3,5-dimethoxybenzylidene)-5-(4-hydroxybenzylidene)cyclopentanone* (**3e**). Yield = 71%; m.p.: 259–261 °C; ^1^H-NMR δ = 10.02 (brs, 1H), 9.02 (brs, 1H), 7.52 (d, *J* = 8.7 Hz, 2H), 7.33 (m, 2H), 6.96 (s, 2H), 6.86 (d, *J* = 8.7 Hz, 2H), 3.82 (s, 6H), 3.08–3.03 (m, 4H); ESI-MS: *m/z* = 350.9 (M^+^−H); Anal. Calc. for C_21_H_20_O_5_: C, 71.58; H, 5.72. Found: C, 71.50; H, 5.74.

*2-(3,5-Di-tert-butyl-4-hydroxybenzylidene)-5-(4-hydroxybenzylidene)cyclopentanone* (**3f**). Yield = 75%; m.p.: 233–235 °C; ^1^H-NMR δ = 10.01 (brs, 1H), 7.66 (s, 1H), 7.51 (d, *J* = 8.7 Hz, 1H), 7.42 (s, 2H), 7.33 (d, *J* = 8.7 Hz, 2H), 6.85 (d, *J* = 8.7 Hz, 2H), 3.03 (s, 4H), 1.42 (s, 18H); ESI-MS: *m/z* = 403.0 (M^+^−H); Anal. Calc. for C_27_H_32_O_3_: C, 80.16; H, 7.97. Found: C, 80.12; H, 8.00. 

*2-(3-Bromo-4-hydroxy-5-methoxybenzylidene)-5-(4-hydroxybenzylidene)cyclopentanone* (**3g**). Yield = 75%; m.p.: 241–243°C; ^1^H-NMR δ = 10.10 (brs, 1H), 10.07 (brs, 1H), 7.52 (d, *J* = 8.7 Hz, 2H), 7.41 (s, 1H), 7.34 (s, 1H), 7.30 (s, 1H), 7.27 (s, 1H), 6.86 (d, *J* = 8.7 Hz, 2H), 3.88 (s, 3H), 3.04 (s, 4H); ESI-MS: *m/z* = 399.0 (M^+^−H); Anal. Calc. for C_20_H_17_BrO_4_: C, 59.87; H, 4.27. Found: C, 59.80; H, 4.29.

*2-(3,5-Dibromo-4-hydroxybenzylidene)-5-(4-hydroxybenzylidene)cyclopentanone* (**3h**). Yield = 69%; m.p.: 286–288 °C; ^1^H-NMR δ = 9.75 (brs, 1H), 8.04 (brs, 1H), 7.83 (s, 2H), 7.53 (d, *J* = 8.7 Hz, 2H), 7.35 (s, 1H), 7.26 (d, *J* = 1.2 Hz), 6.86 (d, *J* = 8.7 Hz, 2H), 3.02 (s, 4H); ESI-MS: *m/z* = 448.7 (M^+^−H); Anal. Calc. for C_19_H_14_Br_2_O_3_: C, 50.70; H, 3.13. Found: C, 50.64; H, 3.14.

*2-(3,4-Dihydroxybenzylidene)-5-(4-hydroxy-3-methoxybenzylidene)cyclopentanone* (**3i**). Yield = 60%; m.p.: >300 °C; ^1^H-NMR δ = 9.62 (brs, 1H), 9.54 (brs, 1H), 9.19 (br, 1H), 7.32 (s, 1H), 7.23 (s, 2H), 7.14 (d, *J* = 8.7 Hz, 1H), 7.09 (s, 1H), 6.98 (d, *J* = 8.7 Hz, 1H), 6.86 (d, *J* = 8.1 Hz, 1H), 6.81 (d, *J* = 8.1 Hz, 1H), 3.82 (s, 3H), 3.03 (s, 4H); ESI-MS: *m/z* = 336.9 (M^+^−H); Anal. Calc. for C_20_H_18_O_5_: C, 70.99; H, 5.36. Found: C, 70.90; H, 5.38.

*2-(2,4-Dihydroxybenzylidene)-5-(4-hydroxy-3-methoxybenzylidene)cyclopentanone* (**3j**). Yield = 60%; m.p.: 223–225 °C; ^1^H-NMR δ = 10.06 (brs, 1H), 9.88 (brs, 1H), 9.62 (brs, 1H), 7.76 (s, 1H), 7.42 (d, *J* = 8.7 Hz), 7.32 (s, 1H), 7.23 (s, 1H), 7.12 (s, 1H), 7.16 (d, *J* = 8.7 Hz, 1H), 6.86 (m, 2H), 3.82 (s, 3H), 3.03 (s, 4H); ESI-MS: *m/z* = 336.9 (M^+^−H); Anal. Calc. for C_20_H_18_O_5_: C, 70.99; H, 5.36. Found: C, 70.86; H, 5.38.

*2-(4-Hydroxy-3,5-dimethoxybenzylidene)-5-(4-hydroxy-3-methoxybenzylidene)cyclopentanone* (**3k**). Yield = 65%; m.p.: 129–131 °C; ^1^H-NMR δ = 9.67 (brs, 1H), 9.06 (brs, 1H), 7.37 (s, 2H), 7.26 (s, 1H), 7.17 (dd, *J* = 8.4, 1.6 Hz, 1H), 6.99 (s, 2H), 6.89 (d, *J* = 8.4 Hz, 1H), 3.83 (s, 6H), 3.11 (m, 4H); ESI-MS: *m/z* = 380.9 (M^+^−H); Anal. Calc. for C_22_H_22_O_6_: C, 69.10; H, 5.80. Found: C, 69.02; H, 5.83.

*1-(4-Hydroxyphenyl)-5-(4-methoxyphenyl)penta-1,4-dien-3-one* (**4a**). Yield = 55%; m.p.: 114–116 °C; ^1^H-NMR δ = 10.04 (brs, 1H), 7.73–7.69 (m, 3H), 7.65–7.59 (m, 3H), 7.15 (d, *J* = 15.0 Hz), 7.09 (d, *J* = 15.0 Hz, 1H), 7.04 (d, *J* = 8.7 Hz, 2H), 6.81 (d, *J* = 8.7 Hz, 2H), 3.80 (s, 3H); ESI-MS: *m/z* = 279.1 (M^+^−H); Anal. Calc. for C_18_H_16_O_3_: C, 77.12; H, 5.75. Found: C, 77.04; H, 5.80.

*1-(4-Hydroxy-3-methoxyphenyl)-5-(4-hydroxyphenyl)penta-1,4-dien-3-one* (**4b**). Yield = 52%; m.p.: 98–101 °C; ^1^H-NMR δ = 10.01 (brs, 1H), 9.62 (brs, 1H), 7. 65–7.60 (m, 4H), 7.34 (s, 1H), 7.19–7.16 (m, 1H), 7.12–7.07 (m, 2H), 6.82–6.80 (m, 3H), 3.83 (s, 3H ESI-MS: *m/z* = 295.0 (M^+^−H); Anal. Calc. for C_18_H_16_O_4_: C, 72.96; H, 5.44. Found: C, 72.84; H, 5.47.

*1-(3,4-Dihydroxyphenyl)-5-(4-hydroxyphenyl)penta-1,4-dien-3-one* (**4c**). Yield = 55%; m.p.: 181–183 °C; ^1^H-NMR δ = 7.69 (d, *J* = 15.3 Hz, 1H), 7.65 (d, *J* = 15.3 Hz, 1H), 7.58 (d, *J* = 8.1 Hz, 2H), 7.15 (s, 1H), 7.08–7.06 (m, 2H), 7.00 (d, *J* = 15.3 Hz, 1H), 6.82–6.80 (m, 3H); ESI-MS: *m/z* = 280.8 (M^+^−H); Anal. Calc. for C_17_H_14_O_4_: C, 72.33; H, 5.00. Found: C, 72.23; H, 5.03.

*1-(4-Hydroxy-3,5-dimethoxyphenyl)-5-(4-hydroxyphenyl)penta-1,4-dien-3-one* (**4d**). Yield = 50%; m.p.: 93–95 °C; ^1^H-NMR δ = 10.04 (brs, 1H), 9.01 (brs, 1H), 7.62 (m, 4H), 7.07 (m, 4H), 6.82 (s, 2H), 3.83 (s, 6H); ESI-MS: *m/z* = 325.1 (M^+^−H); Anal. Calc. for C_19_H_18_O_5_: C, 69.93; H, 5.56. Found: C, 69.86; H 5.59.

*1-(3,5-Dibromo-4-hydroxyphenyl)-5-(4-hydroxyphenyl)penta-1,4-dien-3-one* (**4e**). Yield = 51%; m.p.: 110–113 °C; ^1^H-NMR δ = 10.46 (brs, 1H), 10.06 (brs, 1H), 8.00 (s, 2H), 7.70 (d, *J* = 16.5 Hz, 1H), 7.60 (m, 2H), 7.55 (d, *J* = 16.5 Hz, 1H), 7.30 (d, *J* = 16.2 Hz, 1H), 7.01 (d, *J* = 16.2 Hz, 1H), 6.82 (m, 2H); ESI-MS: *m/z* = 422.9 (M^+^−H); Anal. Calc. for C_17_H_12_Br_2_O_3_: C, 48.15; H, 2.85. Found: C, 48.06; H, 2.88.

*1-(4-Hydroxy-3-methoxyphenyl)-5-(4-methoxyphenyl)penta-1,4-dien-3-one* (**4f**). Yield = 58%; m.p.: 105–107 °C; ^1^H-NMR δ = 9.62 (brs, 1H), 7.58 (m, 3H), 7.15 (m, 5H), 6.72 (m, 3H), 3.80 (s, 6H); ESI-MS: *m/z* = 309.2 (M^+^−H); Anal. Calc. for C_19_H_18_O_4_: C, 73.53; H, 5.85. Found: C, 73.50; H, 5.88.

*1-(3,4-Dihydroxyphenyl)-5-(4-hydroxy-3-methoxyphenyl)penta-1,4-dien-3-one* (**4g**). Yield = 50%; m.p.: 214–216 °C; ^1^H-NMR δ = 7.70 (m, 4H), 7.15 (d, 2H), 7.09 (m, 2H), 6.98 (m, 3H), 6.50 (s, 1H), 3.87 (s, 3H); ESI-MS: *m/z* = 310.8 (M^+^−H); Anal. Calc. for C_18_H_16_O_5_: C, 69.22; H, 5.16. Found: C, 69.15; H, 5.19.

*1-(4-Hydroxy-3,5-dimethoxyphenyl)-5-(4-hydroxy-3-methoxyphenyl)penta-1,4-dien-3-one* (**4h**). Yield = 56%; m.p.: 100–102 °C; ^1^H-NMR δ = 9.75 (s, 1H), 9.65 (s, 1H), 7.63 (m, 2H), 7.17 (m, 4H), 7.07 (s, 2H), 3.83 (d, 9H); ESI-MS: *m/z* = 354.9 (M^+^−H); Anal. Calc. for C_20_H_20_O_6_: C, 67.41; H, 5.66. Found: C, 67.32; H, 5.69.

*1-(3,4-Dihydroxyphenyl)-5-(4-methoxyphenyl)penta-1,4-dien-3-one* (**4i**). Yield = 62%; m.p.: 206–208 °C; ^1^H-NMR δ = 7.68 (d, *J* = 16.5 Hz, 2H), 7.58 (d, *J* = 8.1 Hz, 2H), 7.15 (s, 1H), 7.08–7.06 (m, 2H), 7.01–6.99 (m, 1H), 6.80 (d, *J* = 8.1 Hz, 2H), 6.79–6.77 (m, 1H), 3.87 (s, 3H); ESI-MS: *m/z* = 294.8 (M^+^−H); Anal. Calc. for C_18_H_16_O_4_: C, 72.96; H, 5.44. Found: C, 72.87; H, 5.46.

### 3.3. Tyrosinase Assay

The spectrophotometric assay for tyrosinase was performed according to the reported method [26] with slight modifications. Briefly, all the synthesized compounds were screened for the diphenolase inhibitory activity of tyrosinase using l-DOPA as the substrate. All compounds were dissolved in DMSO. The final concentration of the test solution was 2.0%. Phosphate buffer (pH = 6.8) was used to dilute the DMSO stock solution of test compounds. Thirty units of mushroom tyrosinase (0.5 mg/mL) were firstly pre-incubated with these compounds in 50 mM phosphate buffer (pH = 6.8) at 25 °C for 10 min. l-DOPA (0.5 mM) was then added to the reaction mixture and the enzyme reaction was monitored by measuring the change in absorbance at 475 nm of the formation of l-DOPA chrome for 1 min. The measurement was performed for three times for each concentration and averaged before further calculations. IC_50_ values were determined by the interpolation of the dose–response curves. Kojic acid was used as the standard inhibitor for tyrosinase.

### 3.4. Inhibition Kinetics

The determination of inhibition kinetics was performed by this method: for example, for each of three different inhibitor concentrations of compound **3d** (0.0 μM, 4.06 μM and 16.23 μM, respectively), l-DOPA concentration was varied (5, 10, 15, 20 and 25 μL). Pre-incubation and measurement time was the same as the procedure in section *3.3*. Maximal initial velocity was determined from initial linear portion of absorbance between 0 and 60 s after addition of mushroom tyrosinase. The inhibition type on the enzyme was assayed by Lineweaver–Burk plots, and the inhibition constant was determined by the second plots of the apparent Km/Vm or 1/Vm *versus* the concentration of compound. The same procedure was executed for other compounds.

### 3.5. Acute Toxicity Assays in Mice

The acute toxicity of compound **3d** was examined according to the OECD 423 Guideline for Testing of Chemicals Acute Oral Toxicity – Acute Toxic Class Method procedures [29]. The test procedure was applied with an initial dose at 1,200 mg/kg. Male and female CD-1 mice (six males and six females; 7~8 weeks old) were obtained from Center of Animal Test of the Sun Yat-sen University, housed in the University-approved animal facility in rooms maintained at 22 ± 2 °C with 55%–60% humidity and 12 h photoperiod. After five-day adaptation to laboratory conditions, mice were divided into four equal groups (controls: male, female, and treated: male and female, three animals in each group, equal body mass) and given by gavage either a single dosage of 1,200 mg **3d** (dispersed in 3% Tween 80 aqueous solution)/kg, and equal volumes of distilled water containing 3% Tween 80 for control groups, using a suitably graduated syringe and a stainless steel intubation cannula. Mice had free access to distilled water and commercial standard diet. Animals were observed individually after dosing at least once during the first 30 min, periodically during the first 24 h, with special attention given during the first 4 h, and daily thereafter, for a total of 14 days. All observations were systematically recorded with individual records being maintained for each animal. Individual body weight of animals was determined shortly before the test substance was administered and weekly thereafter. All the animals were sacrificed at the end of the observation period and subjected to a necropsy. The same procedure was performed for compound **3j**.

### 3.6. Molecular Modeling

To date, any attempt to determine experimental X-ray structure of human tyrosinase has failed. To overcome this problem, a homology model was made with the crystal structure of a bacterium tyrosinase taken from *Streptomyces castaneoglobisporus* as template (PDB accession code 2ZWE). The crystal structure of tyrosinase 2ZWE was a complex with caddie protein ORF378 which binded in the active site of tyrosinase. ORF378 was removed [30]. For docking study, all water molecules were removed, and AMBER charges were assigned, orientations of side chain amides were corrected, and hydrogens were added and their positions were optimized by energy minimization using AMBER7 FF99 force field. Compounds **3c** and **3d** were selected and built using the Sybyl 8.0 (Tripos, Inc., St. Louis, MO, USA). After sketching the molecules, Gasteiger-Hückel partial charges were automatically assigned. Energy evaluations were made using the Tripos force-filed. Geometry optimizations were performed using 20 iterations of simplex followed by 500 steps of steepest descent protocol and then 2,000 steps of Powell algorithm’s minimization. The molecular energies of all compounds always converged within the gradient displacement criterion of 0.001 kcal/Å^2^. Docking calculations were performed with Surflex-Dock on the Red Hat workstation. “Protomol” of Surflex-Dock was used to guide the molecular docking. The protomol was defined by setting the threshold value and the bloat value at 0.66 and 2 Å, respectively. The binding pocket of tyrosinase has already been defined with the residues surrounding the dicopper ions since they are implied to the recognition of the l-DOPA substrate [30]. The values of additional starting conformations per molecule and maximum number of poses per ligand were both expanded to 40 to increase the accuracy of binding mode. Other parameters were based on the software default setting. Then the ligands were docked into the active side of tyrosinase under the same condition.

## 4. Conclusions

In summary, we have synthesized various poliphenolic unsymmetrical curcumin analogues (UCAs) using aldol condensation reactions. UCAs containing 4-hydroxyl-substituted phenolic rings were found to be potent tyrosinase inhibitors (with IC_50_ values up to 1.74 μM). The acute toxicity evaluation of **3d** and **3j** showed they were non-toxic in mice dosed at 1,200 mg/kg. This work discloses a rapid assembly of UCAs and in turn. the possibility for numerous types of tyrosinase inhibitor studies. Efforts along these lines are ongoing and will be reported in due course.
